# The influence of sagittal pelvic malrotation on transverse acetabular ligament guided cup orientation: a retrospective cohort study

**DOI:** 10.1186/s12891-021-04391-6

**Published:** 2021-05-28

**Authors:** Tingxian Ling, Zichuan Ding, Mingcheng Yuan, Kai Zhou, Zongke Zhou

**Affiliations:** Department of Orthopedics, Orthopedic Research Institute, West China Hospital, Sichuan University, 37# Guoxue Rd, 610041 Chengdu, China

**Keywords:** Transverse acetabular ligament, Pelvic rotation, Total hip arthroplasty, Primary, Anterior pelvic plane, Cup positioning

## Abstract

**Background:**

Total hip arthroplasty (THA) candidates frequently present pelvic malrotation. The aim of this study is to analyze how pelvic malrotation influence transverse acetabular ligament (TAL) guided cup orientation and investigate whether pelvic malrotation produce different clinical outcomes after THA.

**Methods:**

We retrospectively reviewed a consecutive series of THA patients (144 hips) who use TAL as a guidance for cup positioning from March 2017 to January 2020. The patients were divided into normal pelvis (NP) group and backward pelvis (BP) group by sagittal pelvic malrotation assessed by APPA, the angle between the vertical and the APP on standing lateral pelvic radiographs preoperatively. Cup anteversion and inclination and that out of the safe zones were measured and compared in two groups. The demographic data, clinical results, and complications of patients were also compared.

**Results:**

Backward pelvic malrotation were found in 60.6 % of this cohort of THA candidates. The mean angle of both inclination and anteversion in BP group were significantly larger than that in NP group. The rate of cup for anteversion and inclination above the safe zone in BP group was significantly larger than that in NP group. There were 4 patients in BP group recording anterior hip dislocation after surgery. Other complications were not observed at last follow-up.

**Conclusions:**

Backward pelvis malrotation may increase TAL guided cup inclination and anteversion, which were inclined to became outlier above the safe zone. This likely increase the rates of dislocation after THA. For the patients with pelvis malrotation, cup positioning should be performed individually instead of guided by TAL.

## Background

Optimal positioning of the acetabular component is essential for successful total hip arthroplasty (THA) [1]. Poor cup placement may lead to dislocation, bearing wear, osteolysis, implant impingement, and early failure [2, 3]. Lewinnek et al. reported that acetabular cup placement with 5°-25° of anteversion and an inclination (abduction) angle of 30°-50° is in the safe zone. The dislocation rates were 1.5 % if cups were placed within the safe zone, and increased to 6.1 % when cups were placed outside the safe zone [4]. Patients’ positioning on the operating table, surgeon estimation error, and intraoperative pelvic movement may affect the accuracy of acetabular component orientation [5–7]. Although computer-assisted surgery and positioning patients with great care have the potential to improve accuracy, precise positioning of the acetabular component in the safe zone remains a challenge for the orthopedic surgeon [8, 9].

Using transverse acetabular ligament (TAL) as an intraoperative anatomical landmark for cup position has the benefit of being patient-specific, reproducible, and independent of patient positioning [10]. Since Archbold et al. reported 0.6 % dislocation rate at a minimum 8-month follow-up when TAL was used to determine the anteversion of the acetabular component [11], a number of studies attempted to verify whether TAL can accurately guide cup position [6, 12, 13]. Several studies demonstrated that the inferomedial rim of the cup positioned parallel to TAL may make the cup anteversion within safe zones [14–18]. With respect to cup inclination, Hiddema et al. reported that suitable inclination can be obtained when the inferior rim of the cup is positioned flush with the TAL [10]. However, is really TAL feasible and valid as a guidance for cup positioning?

The anterior pelvic plane (APP) is defined by the plane joining both two anterosuperior iliac spines and the pubic symphysis. The APP is deemed to be both horizontal in the supine position and vertical in standing position in normal individuals [19, 20]. As an anatomical reference, APP is often used to measure the spatial position of the cup after THA, which reflects the anatomical position of the cup [9]. The anatomical position of the cup is the conventional method to describe the cup position [21, 22]. However, referring the cup position to the level ground in standing position after THA reflects the functional position of the cup [20]. The functional position of the cup dictates the outcome following THA in terms of wear and dislocation. The pelvis of normal individual present neutral position in standing posture (APP perpendicular to the ground). In this situation, the functional position and the anatomical position of the cup are equivalent. However, if the pelvis is sagittal malrotation due to degeneration or ankylosing spondylitis in standing position, two of them are different [20]. Therefore, in patients with sagittal pelvic malrotation, despite the correct anatomical cup anteversion and inclination having been obtained intraoperatively by the references of TAL in supine position, the cup anteversion and inclination may be changed once the patient resumes a standing posture. The degree of variation of cup anteversion and inclination in standing posture is mainly related to the level of sagittal malrotation of pelvis [20].

To the best of our knowledge, when use the TAL as a guidance for cup positioning, the influence of sagittal pelvic malrotation on cup anteversion and inclination and for clinical outcomes of THA has been rarely reported. The aim of this study was to analyze how sagittal pelvic malrotation influence TAL guided cup anteversion and inclination and investigate whether it produces different clinical outcomes after THA.

## Methods

A retrospective cohort study was conducted to evaluate the influence of pelvic malrotation on TAL guided cup orientation and clinical outcomes after THA. This study was approved by the Ethics Committee of our hospital. Written informed consent was obtained from all patients. From March 2017 to January 2020, the data of patients who underwent primary THA in our institution were retrospectively analyzed. The inclusion criteria were an age between 20 and 85 years, cup positioning referring to identifiable TAL in operation record, and having weight bearing lateral radiograph of the pelvis (including lateral radiographs of lumbar spine or whole spine) preoperatively and intact pelvic radiographic data at last follow up. Exclusion criteria were a history of any surgery to the hip or spine, severe secondary osteoarthritis due to hip dysplasia or trauma, childhood disease of the hip, previous history of hip tuberculosis or infection, acetabular defect, and tumor of the acetabulum. The patients who had pelvic tilt in the transverse plane on weight bearing radiographs were also excluded. In the end, the data of total 578 patients of primary THA were reviewed. Among them, 132 patients (144 hips) were eligible for the study. There were 70 men and 62 women with an average age of 56.7 years (range, 22 to 83 years) at the time of surgery. The diagnosis of osteonecrosis of femoral head were in 73 patients and osteoarthritis in 59 patients.

All operations were performed by the same surgeon through a posterior approach. After opening the hip articular capsule, the femoral head was resected. The TAL was exposed as a reference for orientation of the acetabular component. If osteophytes and fibrous tissue obscured the inferior portion of the acetabulum, they were carefully removed to expose the TAL. Then, the porous-coated acetabular component (Pinnacle; DePuy, Warsaw, IN) was placed so that the inferior rim of the cup was parallel to the TAL to maintain anteversion, as recommended by Archbold et al. [11], and flush with the TAL to determine inclination, as described by Hiddema et al. [10]. The femoral components were conventionally positioned. The posterior capsule, short external rotators, and piriformis were then repaired. Postoperatively, all patients were placed in an abduction brace and encouraged to perform hip flexion, extension, and abduction while on their bed on the second postoperative day. Partial and full weight bearing walking exercises were sequentially performed.

**Fig. 1:**
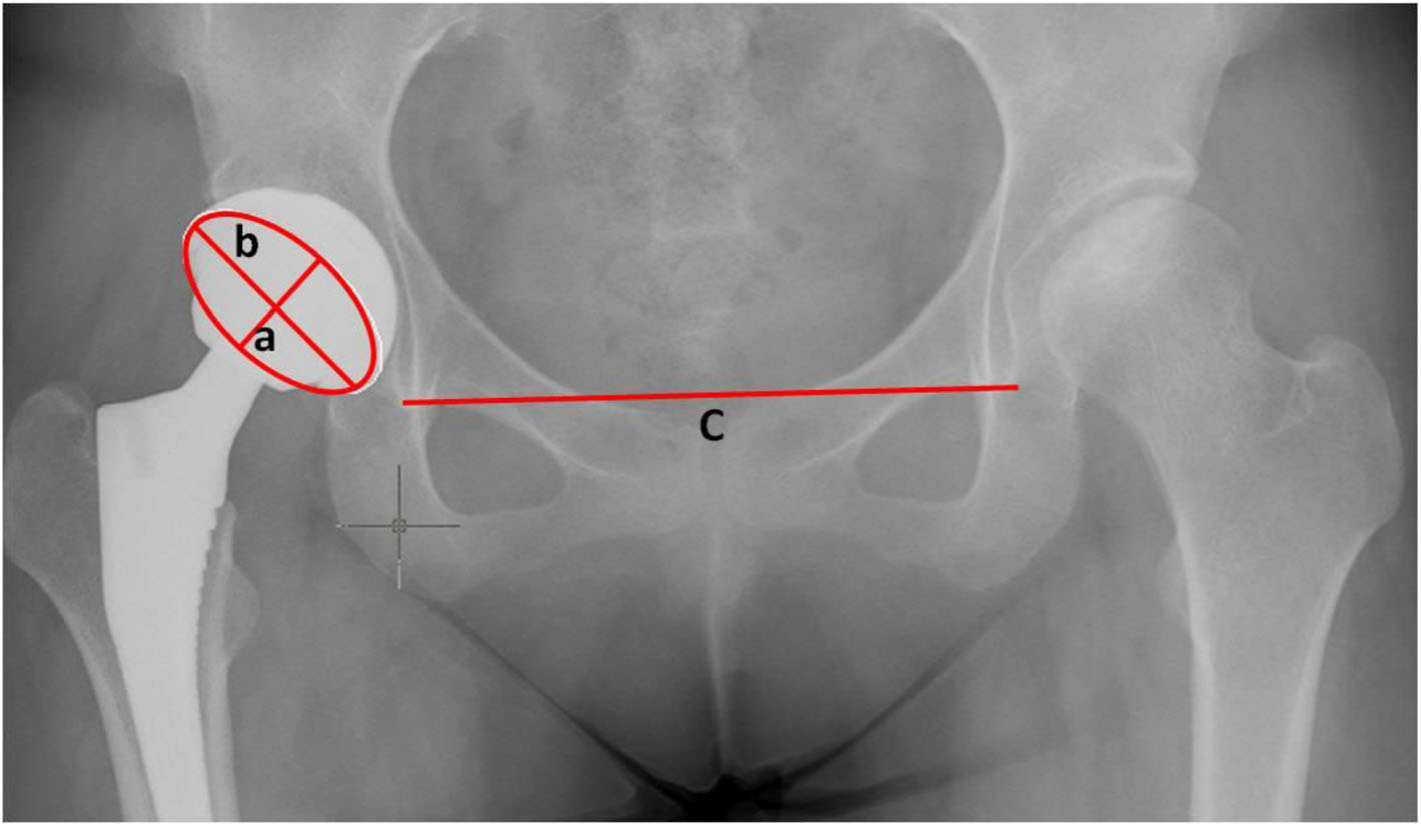
Measurements of the angles of inclination and anteversion on standing anteroposterior pelvic radiographs. The angle of inclination is the angle between the large diameter of the opening ellipse (**b**) and the interteardrop line (**c**). The angle of anteversion is the arcsine of the ratio of the small (**a**) and the large radius (**b**) of the opening ellipse, (= arcsin(a/b))

**Fig. 2:**
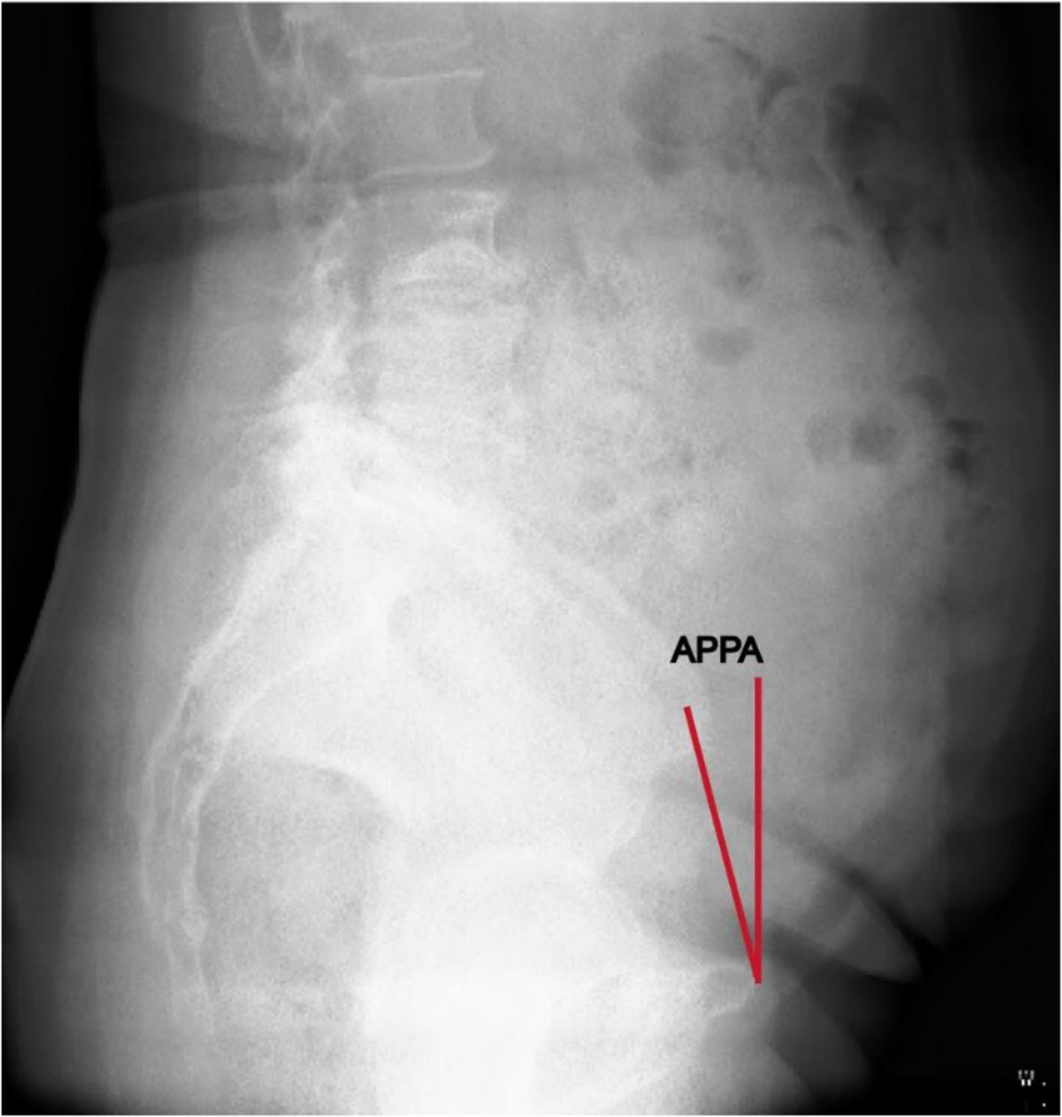
Measurements of the APPA. The APPA was the angle between the line from the pubic tubercles to anterior superior iliac spine and the vertical

Cup anteversion and inclination were measured on standing anteroposterior pelvic radiographs at last follow up. The radiographic data of all patients were reviewed sequentially by two independent orthopedists. The cup inclination was defined as the angle between the inter-teardrop line and the plane of opening of the component [12]. The anteversion angle of the acetabular component was measured according to the technique of Woo et al. [23] (Fig. 1). The safe zones for acetabular cup placement were defined as an anteversion angle of 5°-25° and an inclination angle of 30°-50°. The anterior pelvic plane angle (APPA) was value of the angle between the vertical and the APP. Pelvic rotation was assessed by APPA on standing lateral pelvic radiographs preoperatively (Fig. 2). When the pelvis was in a neutral position, APP was perpendicular to the ground and the APPA was 0°. When the pelvis was backward rotation, the pubic tubercle became anterior to the anterior superior iliac spine and the APPA was positive value. When the pelvis was forward rotation, in contrast, the APPA was negative value. We defined the normal pelvis as the absolute value of APPA less than or equal to 10°. The value of APPA larger than 10° was regarded as backward pelvis and less than − 10° was regarded as forward pelvis. Because the measuring result of APPA were range from − 3.8° to 26.7°, we divided the patients into normal pelvis (NP) group and backward pelvis (BP) group. Cup anteversion and inclination and the rates outside of the safe zones were recorded and compared in two groups. The demographic data and complications of patients were also reviewed.

Measurement data are presented as mean values and standard deviations. The differences in patient demographic characteristics and in radiographic acetabular positioning among the groups were compared using a paired Student’s t-test, while Chi square analysis was used for nominal variables. *P*-values < 0.05 were considered statistically significant. Statistical analyses were performed using IBM SPSS Statistics for Windows, Version 23.0 (IBM Corp., Armonk, NY, USA).

## Results

The average APPA was 13.0° (range, -3.8° to 26.7°). The APPA lager than 10° were found in 80 patients (60.6 %), which were categorized as backward pelvis. The other 52 patients (39.4 %) were normal pelvis. The average follow-up times were 12.8 months (range, 3 to 36 months). The mean cup inclination angle was 38.4 ± 6.9° (range, 20° to 56°) and the mean cup anteversion angle was 21.7 ± 5.6° (range, 6° to 37°). The number of outliers in inclination with respect to the safe zone were 23 hips (16.0 %), and ten of them had an inclination under the safe zone. The number of outliers in anteversion with respect to the safe zone was 38 hips (26.4 %), and all of them had an anteversion above the safe zone. The mean Harris hip scores were 48.7 ± 6.6 preoperatively and significantly improved to 87.8 ± 4.1 at last follow up.

The patients were divided into two groups by APPA. There were 18 males and 34 females with the average age of 51.2 in NP group, and 52 males and 28 females with the mean age of 60.3 in BP group. The mean age in the patient of BP group were significantly larger than that in NP group. The average follow-up times between BP and NP group were no significant difference. The mean angle of both inclination and anteversion in BP group were significantly larger than that in NP group. In addition, the rates of cup for anteversion outside of the safe zone in BP group (32 %, 29 hips) were significantly larger than that in NP group (16.7 %, 9 hips). The rates of cup for inclination outside of the safe zones in BP group (16.7 %, 15 hips) were similar to NP group (14.8 %, 8 hips). However, the rates of cup for inclination above the safe zone (11.1 %, 10 hips) in BP group were significantly larger than that in NP group (5.6 %, 3 hips). The mean Harris hip scores were no significant differences between two groups at last follow up (Table 1).

**Table 1:** Clinical and radiographic data comparison between two groups

	NP Group (*n* = 52, 54hip)	BP Group (*n* = 80, 90hip)	*p*	t
APPA (°)	6.96 ± 3.73	16.55 ± 3.79	0.000	-14.779
Age(yr)	51.15 ± 14.34	60.30 ± 12.10	0.000	-3.942
CAA (°)	20.29 ± 5.27	22.50 ± 5.73	0.022	-2.313
CIA (°)	35.83 ± 7.08	39.90 ± 6.43	0.001	-3.537
HHS(Pre-op)	48.20 ± 6.40	49.06 ± 6.78	0.457	-0.746
HHS(Last-fu)	88.24 ± 4.10	87.48 ± 4.07	0.279	1.087
FUT (m)	12.03 ± 6.40	13.24 ± 8.31	0.361	-0.971

Abbreviations: *NP* normal pelvis, *BP* backward pelvis, *APPA* anterior pelvic plane angle, *CAA* Cup anteversion angle, *CIA* Cup inclination angle, *HHS* Harris hip scores, *FUT* follow-up time

Six patients were recorded hip dislocation after surgery. There were 2 posterior dislocations and 4 anterior dislocations. One patient in NP group is posterior dislocation with FAA of 8°. Among patients in BP group,1 is posterior dislocation with FAA of 11°; 1 is anterior dislocation with FAA of 24°; 3 are anterior dislocation with outlier of FAA above safe zone. The dislocation was treated with closed reduction and plaster immobilization. No further dislocation occurred in subsequent follow-up. Complications of cup loosening, deep venous thrombosis, and hip infection were not observed at last follow-up.

## Discussion

According to the definitions of the Scoliosis Research Society (SRS) [19], APP was assumed to be both horizontal in the supine position and vertical in the standing position. It was initially reported to be independent of sex or age [24]. However, great individual variations in APP in the sagittal inclination were subsequently described [25]. Pinoit et al. [26] observed that APP is vertical in less than 50 % of cases, with a tilt of 5° in 38 % and of 10° in 13 %. Forward pelvic tilt can be directly induced by fixed flexion of the hips or knees. Backward pelvic tilt, generally in the elderly, is related to a low value of lumbar lordosis insufficient to compensate for kyphosis. Lee et al. [27] compared the spinopelvic sagittal parameters between the osteoporosis group with an average age of 72.4 years and the control group with an average age of 42.7 years. They found that the pelvic tilt in the control group was significantly lower than in the osteoporosis group. In our study, the average age in BP group was larger than in NP group, similar to the study by Lee et al., meaning that the backward pelvic tilt may result from degeneration with increased age. The degree of pelvic tilt is evaluated by the APPA. According to the review of the literature, pelvic tilt in THA candidates has been infrequently reported. We found that 60.6 % of THA candidates in this cohort have backward pelvic tilt.

We had strict patient inclusion and exclusion criteria. The patients in whom both cup positioning referring to identifiable TAL in operation record and having weight bearing lateral radiograph of the pelvis preoperatively and intact pelvic radiographic data at last follow up were included. In addition, rotation of the pelvis in the coronal plane will result in inaccurate sagittal plane assessment and can affect abduction as well. Therefore, the patients with an acetabular defect, severe DDH, or childhood disease of the hip leading to pelvic inclination in the coronal plane were excluded. Moreover, degeneration is emphasized as the main factor affecting the pelvic tilt in our study. Thus, patients with a history of surgery to the hip or spine or a previous history of hip tuberculosis or infection affecting the pelvic tilt were also excluded.

In the current study, we found that inclination in 16.0 % patients and anteversion in 26.4 % patients were outside of the safe zones. Miyoshi et al. [15] reported that anteversion in 10.6 % patients was outside of the safe zone using the TAL as a reference. Archbold et al. [11] showed the rate of dislocation after THA was reduced to 0.6 % using the TAL as a guide for cup positioning. However, anteversion outside of the safe zones was not reported. Meermans et al. [14] reported that anteversion in 0 % of components and inclination in 20 % of components were outside of the safe zones in the TAL group. Epstein et al. [12], who considered that the TAL was not a helpful guide in orienting the acetabular component, reported that anteversion in 41 % components and inclination in 17 % components were outside of the safe zones in the TAL group. These studies above reported that the anteversion and inclination outside of the safe zones had a large difference. We think that the following several reasons may help to explain this. First, surgeons in studies above position patients lying on X-ray radiographic table and adjust APP parallel with the horizontal plane to take standard postoperative anteroposterior radiographs of the pelvis to measure the anatomical cup orientation [4, 12, 14, 15, 17]. However, radiographs of the pelvis were taken in the standing position to measure the functional cup position in current study. In patients with sagittal malrotation of pelvis, elderly patients of osteoarthritis and ankylosing spondylitis patients, APP is not vertical in the standing position [20, 28]. Therefore, the functional position and the anatomical position of the cup are different in such patients. Cup position measured by APP in supine position would be changed by sagittal pelvic malrotation once the patient resumes a standing posture. In addition, the anatomic structure difference of the TAL and the pelvis also likely influence the measurement of cup position [29].

In order to further clarify the influence of pelvic malrotation on TAL guided cup anteversion and inclination, we divided the patients into two groups by 10° of APPA. The grouping bases were discussed as follows. Legaye et al. [21] analyzed the distribution range of APPA in healthy volunteers with the average age of 44 and in subjects suffering from low back pain with the average age of 61. They reported the APPA in all healthy volunteers were in the range of -10° to 10°. However, in subjects suffering from low back pain, 23 % of APPA were out the range of -10° to 10°. In addition, Tang et al. [20] explore the relationship between functional cup position and pelvic malrotation on a three-dimensional computer model. The cup was implanted by the conventional method that the APP were parallel to the operating table and then inserted with 45° of inclination and 20° of anteversion. When the pelvis resumes a neutral position with the pelvic rotation, the functional cup inclination and anteversion were changed. With the pelvis anteverted to 10°, the functional cup inclination and anteversion will increased to 50° and 25° respectively, which are upper normal limits of safe zone. Therefore, we consider that the value of APPA range from − 10° to 10° were defined as normal pelvis, and greater than 10° were regarded as backward pelvis.

After grouping by APPA, we found that the mean angle of both inclination and anteversion in BP group were significantly larger than that in NP group. Moreover, the rates of cup for anteversion and inclination above the safe zone in BP group were significantly larger than that in NP group. Our results were similar to those of Tang et al. [20] reported. This means that the backward tilt of pelvis probably resulted in the increase of TAL guided anteversion and inclination, which were inclined to became outlier above the safe zone. The cup was positioned inside of safe zones with the anatomical reference of TAL in supine position. However, because of backward tilt of pelvis, the anteversion and inclination for the same patient were probably outside of the safe zones after standing up. This suggest that the evaluation of pelvis rotation in sagittal plane should be done by weight bearing lateral radiograph of the pelvis before THA. Smaller anatomical anteversion and inclination of the cup should be performed in the patient with backward pelvis to obtain a suitable anteversion and inclination on standing position. Although the rates of cup for anteversion and inclination outside of safe zones were high in BP group, the hip function scores between two groups were no significant difference at last follow-up. In the aspect of complications, both of two groups were not found cup loosening, deep venous thrombosis, and hip infection at last follow-up. However, patients with anterior dislocation due to overlarge cup anteversion angle were all in BP group. This indicate that backward tilt of pelvis may increase the rates of anterior hip dislocation after THA. Specific anteversion and inclination according to pelvic malrotation should be performed for these patients to prevent hip dislocation instead of guided by TAL. There were several limitations of the current study. First, this was a retrospective and single-center study. The number of subjects was relatively limited. A prospective and multi-center study with a larger sample size is needed to further evaluate the influence of backward pelvic tilt on cup anteversion and inclination. In addition, the average follow-up times were only 12.8 months in current study. The long-term follow-up results of the influence on hip functions and complications by backward pelvic tilt need to further evaluate in subsequent studies. Moreover, with the increase of age and the degree of lumbar degeneration, whether the backward pelvic tilt and the rate of cup for inclination and anteversion outside of safe zone will be increased need to be further clarified.

## Conclusions

Backward pelvis malrotation may increase TAL guided cup inclination and anteversion, which were inclined to became outlier above the safe zone. This likely increase the rates of dislocation after THA. Thus, we suggest that sagittal pelvic malrotation in standing position need to be assessed preoperatively. For patients with pelvis malrotation, cup positioning should be performed individually instead of guided with TAL.

## Data Availability

The datasets generated during and/or analyzed during the current study are available from the corresponding author on reasonable request.

## Abbreviations

THA: Total hip arthroplasty
TAL: Transverse acetabular ligament
HHS: Harris hip score
NP: Normal pelvis
BP: Backward pelvis
APPA: Anterior pelvic plane angle
CIA: Cup inclination angle
CAA: Cup anteversion angle
FUT: Follow-up time
